# Therapeutic Strategies against Epstein-Barr Virus-Associated Cancers Using Proteasome Inhibitors

**DOI:** 10.3390/v9110352

**Published:** 2017-11-21

**Authors:** Kwai Fung Hui, Kam Pui Tam, Alan Kwok Shing Chiang

**Affiliations:** 1 Department of Paediatrics and Adolescent Medicine, Li Ka Shing Faculty of Medicine, The University of Hong Kong, Queen Mary Hospital, Pokfulam, Hong Kong, China; kfhui@hku.hk (K.F.H.); edwintkp@connect.hku.hk (K.P.T.); 2 Center for Nasopharyngeal Carcinoma Research, The University of Hong Kong, Hong Kong, China

**Keywords:** Epstein-Barr virus, proteasome inhibitor, apoptosis, cell cycle, lytic reactivation, Epstein-Barr virus nuclear antigen (EBNA)-3C

## Abstract

Epstein-Barr virus (EBV) is closely associated with several lymphomas (endemic Burkitt lymphoma, Hodgkin lymphoma and nasal NK/T-cell lymphoma) and epithelial cancers (nasopharyngeal carcinoma and gastric carcinoma). To maintain its persistence in the host cells, the virus manipulates the ubiquitin-proteasome system to regulate viral lytic reactivation, modify cell cycle checkpoints, prevent apoptosis and evade immune surveillance. In this review, we aim to provide an overview of the mechanisms by which the virus manipulates the ubiquitin-proteasome system in EBV-associated lymphoid and epithelial malignancies, to evaluate the efficacy of proteasome inhibitors on the treatment of these cancers and discuss potential novel viral-targeted treatment strategies against the EBV-associated cancers.

## 1. Introduction

Epstein-Barr virus (EBV) is a gamma-herpesvirus which infects more than 90% of the world’s population. It is closely associated with several lymphomas (endemic Burkitt lymphoma (BL), Hodgkin lymphoma and nasal NK/T-cell lymphoma) and epithelial cancers (nasopharyngeal carcinoma (NPC) and gastric carcinoma). Since proteasome is crucial for cellular homeostasis, disruption of its function is found to be present in numerous cancers, including virus-associated cancers [1,2]. It has been shown that manipulation of the function of ubiquitin-proteasome system by EBV (and another gamma-herpesvirus, Kaposi’s sarcoma-associated herpesvirus (KSHV)) is indispensable for the survival and replication of the viruses in the infected cells. The viruses can express both lytic and latent proteins to either inhibit the proteasomal degradation of important viral proteins or promote the degradation of unwanted cellular proteins in the virus-associated cancers [3,4,5]. For instance, the disruption of PML (promyelocytic leukaemia) nuclear bodies and subsequent inhibition of ubiquitin-proteasome degradation system by EBV genes (BZLF1, BRLF1, BDLF1, BLLF2, BFLF2, BPLF1, BNRF1, latent membrane protein (LMP)-1, EBV nuclear antigen (EBNA)-1 and EBNA-3B), KSHV genes (replication and transcription activator (RTA), viral interferon regulatory factor (vIRF)-3, open reading frame (ORF)-64 and ORF-75) and mouse hepatitis virus (MHV)-68 genes (ORF-64 and ORF-75C) are demonstrated to be essential for evading the innate immune response during early infection stage [6]. Such immune evasion mechanisms during early viral infection are comprehensively reviewed by Full et al. in 2017 [6]. In this review, we focus on how EBV proteins utilize the ubiquitin-proteasome system to promote degradation of cellular proteins for their survival and the potential usage of proteasome inhibitors in the treatment of EBV-associated malignancies. Specifically, the functions of the key viral proteins (BDLF3, EBNA-1, LMP-1 and EBNA-3C) involved in the manipulation of ubiquitin-proteasome system for inhibition of cell cycle checkpoint, apoptosis and immune surveillance in the EBV-associated malignancies are summarized. The efficacy of proteasome inhibitors on the treatment of EBV-associated malignancies and potential novel viral-targeted treatment strategies using proteasome inhibitors against EBV-associated cancers are discussed.

## 2. The Ubiquitin-Proteasome System

### 2.1. Structure and Function of Proteasome

**Figure 1:**
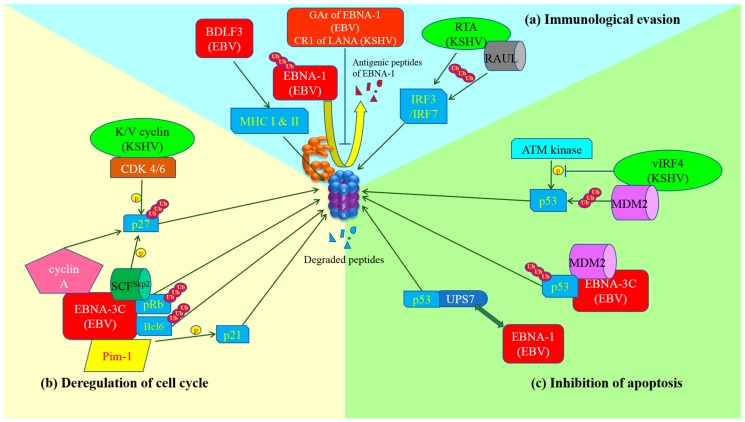
Schematic diagram of exploitation of ubiquitin-proteasome system by gamma-herpesviruses and the development of cancer hallmarks. (**a**) Immunological evasion: GAr domain of Epstein-Barr virus (EBV) nuclear antigen (EBNA)-1 or Kaposi’s sarcoma-associated herpesvirus (KSHV) central repeat (CR)1 of latency-associated nuclear antigen (LANA)inhibits proteasome so as to prevent the proteolysis of EBNA-1 and the production of its antigenic peptides for major histocompatibility complex (MHC) class I presentation. BDLF3 promotes internalization and proteasomal degradation of MHC molecules. As a result, cytotoxic T lymphocytes (CTLs) are not able to detect and kill the latent viruses-infected cells. Replication and transcription activator (RTA) (KSHV) itself or through stabilization of RTA-associated ubiquitin ligase (RAUL) facilitates the ubiquitination and proteasomal degradation of interferon regulatory factor (IRF)3 and IRF7, which are important for innate immunity; (**b**) Deregulation of cell cycle: EBNA-3C can stably interact with pRb and recruit SKP1-Cul1-F-box protein (SCF)^Skp2^ E3-ubiquitin ligase to promote degradation of pRb. Thus, E2F is released and activates the transcription of cyclin-dependent kinases for cell cycle progression. Moreover, EBNA-3C also physically interacts with and degrades Bcl-6 through ubiquitin-specific protease (UPS) but the ligase that facilitates the ubiquitination is still under investigation. EBNA-3C interacts with Pim-1 which enhances the phosphorylation of p21^WAF1^ and promotes proteasomal degradation of p21^WAF1^. The association of SCF^Skp2^ with EBNA-3C or cyclin-dependent kinase (CDK)4/6 with K/V cyclin (KSHV) increases phosphorylation and proteasomal degradation of p27^KIP1^. Additionally, stabilization of cyclin A by EBNA-3C also promotes degradation of p27^KIP1^ through UPS. In summary, gamma-herpesviruses possess multiple mechanisms to assist the infected cells to bypass cell cycle checkpoints for proliferation; (**c**) Inhibition of apoptosis: EBNA-1 displaces p53 in the interaction with ubiquitin-specific protease 7 (USP7), resulting in destabilization of p53 and its degradation by proteasome. On the other hand, MDM2 E3-ubiquitin ligase is recruited and stabilized by EBNA-3C or vIRF4 (KSHV), leading to ubiquitination and proteasomal degradation of p53. Viral interferon regulatory factor (vIRF)4 also inhibits the phosphorylation of p53 by ataxia-telangiectasia mutated (ATM) kinase upon DNA damage response, causing destabilization and proteasomal degradation of p53.

The 26S proteasome is composed of 19S regulatory particle (RP) and 20S core particle (CP), resulting in 26 Svedberg units in sucrose gradient sedimentation. α- and β-subunits constitute the barrel-shaped 20S CP. Two sets of seven α-subunits at both ends form the end rings, whereas two sets of seven β-subunits in the middle form the central rings of 20S CP (Figure 1). The N-termini of β1, β2 and β5 are the active sites responsible for the proteolysis of substrates. β1, β2 and β5 are responsible for proteolytic cleavage of post-glutamylpeptidyl-hydrolyzing (PGPH), trypsin-like and chymotrypsin-like substrates, respectively. Virtually all peptide bonds can be hydrolyzed by these three proteolytic subunits. [7,8]. On the other hand, 19S RP is a proteasome activator (PA) which facilitates the recognition of targeted substrates with polyubiquitin modification and insertion of substrates into the central cavity of 20S CP through adenosine triphosphate (ATP)-dependent mechanism. The ubiquitin molecules are subsequently recycled from the modified proteins by deubiquitinating enzymes (DUBs) [9].

### 2.2. Proteasomal Degradation Mediated by Ubiquitination

Most of the protein degradations catalyzed by proteasome are initiated by polyubiquitination of the targeted proteins. This process is a covalent attachment of ubiquitin, which consists of 76 amino acids, to the substrates and assembles a chain with at least four ubiquitin molecules. First, ubiquitin (Ub) is activated by one of the two ubiquitin-activating enzymes, known as E1, through ATP-dependent mechanism, forming a thioester bond (S) between the cysteine residue at E1 and the carboxyl terminus of ubiquitin. The activated ubiquitin in this Ub-S-E1 intermediate is then transferred by one of the 38 ubiquitin conjugating enzymes, known as E2, forming another intermediate, Ub-S-E2, which serves to bring ubiquitin to the targeted substrate and the ubiquitin-protein ligase, E3 [10]. Multiple ubiquitin molecules are added on the targeted protein to form an ubiquitin chain which is recognized by 19S RP to activate proteolysis [11,12,13]. There is also an E4 ubiquitination factor which is responsible for elongation of the ubiquitin chain in some cases [14].

## 3. Interaction of EBV with Ubiquitin-Proteasome System in Host Cells

As mentioned above, manipulation of the normal function of ubiquitin-proteasome system by EBV (and KSHV) is indispensable for the replication of the viruses and survival of the virus-infected cells. It has been shown that some viral proteins of EBV share similar functions with those of KSHV in the manipulation of ubiquitin-proteasome system. Therefore, understanding the mechanism of the interaction between the viral proteins of KSHV and the proteasome may further provide insight into EBV-proteasome interaction. In this section, the functions of the key viral proteins involved in the manipulation of ubiquitin-proteasome system for inhibition of immune surveillance, cell cycle checkpoint and apoptosis in the EBV- and KSHV-associated malignancies are summarized (refer to Figure 1 & Table 1).

### 3.1. Immunological Evasion

Ubiquitin-proteasome system is important for generation of antigenic peptides of viral proteins for presentation to cytotoxic T cell (CTL) in the context of major histocompatibility complex (MHC) class I [15]. However, EBV develops strategies to evade immune detection and elimination through inhibition of ubiquitin-proteasome system. In latently infected B cells, the glycine-alanine repeat (GAr) domain of EBNA-1 was shown to be the key element in the inhibition of proteasomal processing of EBNA-1 into antigenic peptides and hence prevented MHC class I presentation to CTL and subsequent clearance of the infected cells [16]. This type of immunological evasion is also observed in KSHV-infected cells. The latency-associated nuclear antigen (LANA) of KSHV, which is functionally similar to EBNA-1 though without any sequence homology, prevented the generation of antigenic peptides for MHC class I presentation by inhibiting proteasome [17].

It was also confirmed that BDLF3, a late lytic protein, triggered the internalization and proteasomal degradation of both MHC class I and II molecules on the surface of infected cells, resulting in impairment of immune recognition of the EBV-infected cells by antigen-specific CD8^+^ and CD4^+^ cells [18]. Interestingly, K3 and K5 proteins encoded by KSHV also served similar function as stimulating polyubiquitination and endocytosis of MHC class I molecules from the surface of infected cells. However, unlike BDLF3, the MHC proteins were degraded by lysosome rather than proteasome [19].

In addition, BNRF2, EBNA1 and BZLF1 proteins of EBV could also disrupt the PML-nuclear bodies and subsequently evade the intrinsic antiviral response via proteasome-dependent and independent mechanisms [6]. On the other hand, RNA transcriptional activator (RTA) of KSHV could act as a viral E3-ubiquitin ligase and stabilize the RTA-associated ubiquitin ligase (RAUL) of the host cells. As a result, RTA could facilitate the proteasomal degradation of Interferon regulatory factors (IRF3 and IRF7), hence evading the innate immunity [20,21].

### 3.2. Modulation of Cell Cycle Checkpoints

The major EBV protein that overrides cell cycle regulation is Epstein-Barr nuclear antigen-3C (EBNA-3C) which is expressed in Type III latency. EBNA-3C is known to be crucial for transformation, proliferation and survival of infected B cells. It was found to epigenetically repress the transcription of p16^INK4A^, which is a cyclin-dependent kinase (CDK) inhibitor, hence stabilizing the cyclin D1/CDK6 complex, which increases the phosphorylation and ubiquitin-dependent proteasomal degradation of pRb and allows the cells to enter G1 phase [22]. In addition, the ubiquitin-proteasome degradation of pRb is also enhanced by recruitment of the SKP1-Cul1-F-box protein (SCF^Skp2^) E3-ubiquitin ligase when EBNA-3C stably associates with pRb [4,23]. The transcription factors of E2F family are released from pRb and activate transcription of cyclin/CDK complexes such as cyclin D1/CDK4/6 and cyclins A and E/CDK2, promoting cell cycle progression from G0 to G1 and from G1 to S phase [23].

EBNA-3C also stabilizes proto-oncogene serine/threonine-protein kinase (Pim-1) by physical interaction and enhances its phosphorylation activity on CDK inhibitor p21^WAF1^, leading to proteasomal degradation of p21^WAF1^ and escape of G1-S phase cell cycle arrest [24]. In addition, EBNA-3C activates Skp2, which is one of the two integral proteins of SCF^Skp2^ E3-ubiquitin ligase, leading to phosphorylation and subsequent ubiquitin-dependent proteasomal degradation of another CDK inhibitor p27^KIP1^. This releases the inhibitory effect of p27^KIP1^ to cyclin A/CDK2 complex, stimulating the entry into S phase [25]. Moreover, EBNA-3C specifically interacts with and activates ubiquitination and proteasomal degradation of Bcl-6. As a result, the normal suppression of cyclin D1 by Bcl-6 is abrogated, promoting G1-S phase transition [26]. Since cyclin A is also essential for the initiation of mitosis, the stabilization of cyclin A by EBNA-3C might also assist EBV-infected cells to progress to M phase [27,28].

Of note, p27^KIP1^ is also directed to proteasomal degradation in primary effusion lymphoma (PEL) by KSHV viral proteins, K-cyclin and V-cyclin, which are homologs of human cyclin D2 and form a complex with CDK4/6. The Thr48 residue of p27^KIP1^ is phosphorylated by K/V-cyclin-CDK6 complex, resulting in polyubiquitination and proteasomal degradation of p27^KIP1^ [29,30].

### 3.3. Inhibition of Apoptosis

Several tumor viruses such as EBV, KSHV, hepatitis B virus (HBV) and human papillomaviruses (HPV) were found to promote oncogenesis through prevention of apoptosis coupled with deregulation of the cell cycle. Numerous pro-apoptotic tumor suppressors are suppressed by oncogenic proteins or degraded by proteasome [31]. EBV depends on proteasomal processing of nuclear factor kappa-light-chain-enhancer of activated B cells (NF-κB) precursor p100 into p52 to activate the non-canonical NF-κB pathway, which in turn promotes the expression of inhibitor of apoptosis proteins (IAP), including XIAP, cIAP-1 and c-IAP-2 for cell survival through the action of LMP-1 [32]. EBNA-1 stably associates with herpesvirus associated ubiquitin-proteasome system (HAUSP), also known as ubiquitin-specific protease 7 (USP7), by binding to the same pocket that p53 binds. Thus, the deubiquitination of p53 by USP7 is blocked, leading to its degradation [33]. EBNA-3C protects MDM2 from ubiquitination and proteasomal degradation through physical interaction between its N-terminal 130–190 amino acid residues and the central acidic domain of MDM2, further suppressing p53 [34]. On the other hand, EBNA-3C stimulates proteasomal degradation of Bcl-6, releasing the anti-apoptotic protein, Bcl-2 from suppression by Bcl-6 [26]. Some viral proteins encoded by KSHV can also inhibit apoptosis, for instance, vIRF4 which inhibits the phosphorylation of Ser 15 in p53 by ataxia-telangiectasia mutated (ATM) kinase, leading to destabilization as well as proteasomal degradation of p53 [35]. vIRF4 also stabilizes MDM2 E3-ubiquitin ligase, promoting proteasomal degradation of p53 [36].

## 4. Rationale of Using Proteasome Inhibitor to Treat EBV-Associated Cancers

### 4.1. Overview of Proteasome Inhibitors

Proteasome inhibitors are primarily designed to target on β5 of the 26S proteasome [37,38]. The proteasome inhibitors exert inhibition via either non-covalent (reversible) or covalent (irreversible) binding to the active sites of 20S core particle. There are 8 major structural classes of proteasome inhibitors which include aldehydes, boronates, epoxyketones, α-ketoaldehydes, β-lactones, vinyl sulfones, syrbactins and bacteria specific oxatiazol-2-ones. Some of the proteasome inhibitors, including those in the classes of boronates, epoxyketones and β-lactones, are currently the Food and Drug Administration (FDA)-approved for treatment of various types of cancers or are tested in clinical trials (Table 2). Bortezomib was the first proteasome inhibitor approved by FDA in 2003 for the treatment of multiple myeloma and mantle cell lymphoma [39]. Proteasome inhibitors were demonstrated to inhibit cell growth and promote cell death in a variety of cancers such as multiple myeloma, anaplastic thyroid cancer, colon cancer, pancreatic cancer [38,40,41]. Several possible mechanisms were suggested to explain the anti-cancer effects mediated by proteasome inhibitors. Inhibition of NF-κB signaling pathway was believed to be one of the key anti-cancer mechanisms. Proteasome inhibitors such as bortezomib could significantly suppress the expression of NF-κB and its downstream signaling in various types of cancer cell lines. Another possible cell death mechanism induced by proteasome inhibitors involved phorbol-12-myristate-13-acetate-induced protein 1 (PMAIP1), a pro-apoptotic player of Bcl-2 family. Upon suppression or activation of such signaling pathways, both intrinsic mitochondrial and extrinsic (death receptor) apoptotic pathways were induced. Various cell cycle regulatory proteins such as p21^WAF1^ and p27^KIP1^ and pro-apoptotic proteins such as Bax and Bid were affected by proteasome inhibitors.

### 4.2. Effect of Proteasome Inhibitors on Cell Cycle of EBV-Associated Malignancies

EBV expresses different latency patterns in different EBV-associated malignancies [42]. During primary infection of B cells, EBV expresses the full panel of transforming proteins which drive the continuous proliferation of B cells via modulation of cell cycle checkpoints. EBNA-3 proteins, in particular -3A and -3C, are known to play a role in the manipulation of the cell cycle in EBV-infected B cells. In 1994, Allday et al. first discovered the cell cycle regulatory property of EBNA-3C by showing that expression of wild-type EBNA-3C was able to rescue the G1 arrest in EBNA-3C mutated cells [43]. EBNA-3C was later found to promote G1-S transition by enhancing pRb degradation [4], stabilizing cyclin D1 [44] and facilitating p27^KIP1^ degradation [4]. Besides, EBNA-3C could co-operate with EBNA-3A to epigenetically repress the INK4 family of the CDK inhibitors (e.g., p14 and p16^INK4A^) to support the continuous proliferation of lymphoblastoid cell lines (LCLs) [45,46,47,48,49]. EBNA-3A and -3C could also abrogate the G2/M checkpoint for the survival of EBV-transformed B cells in response to various cytotoxic stresses [50,51,52,53,54].

Proteasome inhibitors were shown to induce G2/M arrest through generation of reactive oxygen species (ROS) and up-regulation of cell cycle regulators such as p21^WAF1^ and p27^KIP1^ in certain cancer types [55,56,57]. In our previous study, we observed that bortezomib could induce G2/M arrest in P3HR1 Wp-restricted BL cells [58]. However, the percentage of cells in G2/M phase was much lower in LCLs which expressed higher level of EBNA-3 proteins [58]. Similarly, EBNA-3 proteins were reported to disrupt G2/M arrest induced by azelaic bishydroxamine in LCLs [50]. We postulated that EBNA-3C would be the major protein involved in the disruption of G2/M arrest because of its ability to modulate the expression of various cell cycle regulatory proteins. [42,45,46,47,48] For instance, EBNA-3C was shown to stabilize cyclin A and assist the EBV-infected cells to progress to M phase [27,28]. Interestingly, bortezomib could also induce the expression of pRb and p21^WAF1^ which were downregulated by ubiquitin-dependent proteasomal degradation [4,22,23,24,25].

### 4.3. Effect of Proteasome Inhibitors on Apoptosis of EBV-Associated Malignancies

EBV latent proteins, including LMP-1, EBNA-3A and EBNA-3C, were shown to be oncogenic and might contribute to the resistance of EBV-associated cancers to apoptotic inducers. LMP-1 is a well characterized viral protein which possesses strong oncogenic activity through activation of NF-κB pathway in both BL and NPC [59,60]. We had shown that bortezomib, which is known to inhibit the NF-κB pathway, could induce apoptosis in BL, gastric carcinoma and NPC [58,61]. Another study showed that bortezomib could inhibit the proteasome-mediated activation of p52 and thus suppress the anti-apoptotic function initiated by the non-canonical NF-κB pathway in EBV-transformed LCLs [62]. EBV-positive BL cells of type III latency were found to be more resistant to killing by nocodazole or taxol when compared with EBV-negative or latency I BL cells [63]. Wp-restricted BL cells were also more resistant to the treatment with ionomycin or anti-IgM when compared with latency I BL cells [64]. Further studies showed that EBV offered essential anti-apoptotic effects to both Wp-restricted BL and post-transplant lymphoproliferative diseases (PTLD) cells but not to latency I BL cells which do not rely on EBV for their survival [65]. Interestingly, we found that bortezomib could induce more potent apoptosis in LCLs when compared with BL cells, suggesting a potential specific effect of bortezomib in disrupting the survival function conferred by EBV in the LCLs [58]. The mechanisms of induction of apoptosis would be related to the disruption of downstream survival signaling of EBNA-3C through its effects on p53, MDM2 and Bcl-6 (as discussed in Section 3.3) [34,66,67].

### 4.4. Reactivation of Viral Lytic Cycle by Proteasome Inhibitors

Bortezomib was reported to reactivate the lytic cycle of EBV in EBV-associated BL cells [62,68]. The EBV lytic reactivation was shown to be related to the induction of CCAAT/enhancer-binding proteinβ (C/EBPβ) and unfolded protein response [68]. Induction of EBV lytic cycle by bortezomib could activate the radioisotope [^125^I]2′-fluoro-2′-deoxy-β-d-5-iodouracil-arabinofuranoside to selectively suppress the growth of Burkitt lymphoma xenografts in severe combined immunodeficiency (SCID) mice [69]. Similarly, induction of KSHV lytic cycle in xenograft model of primary effusion lymphoma (PEL) had also been reported [70]. Recently, a study found that bortezomib could reactivate the lytic cycle of both EBV and KSHV from latency in several PEL and BL cell lines through activation of c-Jun N-terminal kinase (JNK), endoplasmic reticulum (ER) stress and autophagy [71]. However, such viral lytic cycle reactivation by bortezomib had not been observed in a panel of EBV-associated tumor cell lines we tested. In contrast, our results showed that bortezomib suppressed the lytic cycle reactivation by histone deacetylase (HDAC) inhibitors in EBV-associated cancer cell lines [61,72]. Lymphoid cells were more refractory to the induction of EBV lytic cycle than epithelial cells. Indeed, we found that HDAC inhibitors and some novel compounds, which could reactivate EBV lytic cycle in nasopharyngeal carcinoma and gastric carcinoma cells, could only induce low expression level of EBV lytic proteins in one out of six lymphoid cell lines [58,73,74,75,76].

### 4.5. Effect of Proteasome Inhibitors on Immune Evasion

EBV proteins such as BZLF1, BNRF2, BDLF3 and EBNA-1 utilize the proteasomal system to facilitate the virus to escape from human immune surveillance [6]. However, the effect of proteasome inhibitors on immune evasion of EBV-associated malignancies remains largely unknown. Recently, a study showed that proteasome inhibitors could increase the expression of lymphocyte stimulatory cytokine such as interleukines IL-2, IL-12 and IL-15 and activate the p38 and Akt pathways in tumor-infiltrating CD8^+^ T cells [77]. Interestingly, another recent study showed that bortezomib could inhibit the downstream signaling of indoleamine 2,3-dioxygenase, a major inducer of immune tolerance during tumor development, through suppression of signal transducer and activator of transcription 1 (STAT1) in NPC cells [78]. More investigations on the effect of proteasome inhibitors on immune evasion mechanisms of EBV are needed to further develop proteasome inhibitors as a new class of therapeutic agents against EBV-associated cancers.

## 5. Potential Novel Viral-Targeted Strategies against EBV-Associated Cancers by Combination of Proteasome and Histone Deacetylase (HDAC) Inhibitors

Proteasome inhibitors were reported to have synergistic effects when co-administered with other types of anti-tumor compounds [38]. The most well recognized combination would be the combination of bortezomib and a HDAC inhibitor, suberoylanilide hydroxamic acid (SAHA). Combined bortezomib and SAHA was shown to be effective in the treatment of hematologic malignancies such as multiple myeloma [79], mantle cell lymphoma [80], cutaneous T-cell lymphoma [81] and leukemia [82,83]. The drug combination induced cancer cell death through caspase activation [80,82,83,84], generation of reactive oxygen species (ROS) [79,80,81,82,85], enhanced histone acetylation [86,87], aggresome disruption [88], NF-κB inactivation [79,80,82,83,85], p53 activation, p21^WAF1^ up-regulation [79,81,82,84], c-Jun NH2-terminal kinase (JNK) activation [79,83] and mitochondrial membrane dysfunction [79,82,83,84]. The anti-tumor effects of combination of proteasome and HDAC inhibitors on EBV-associated epithelial and lymphoid malignancies will be discussed in this section.

### 5.1. Combination of Proteasome and HDAC Inhibitors on EBV-Associated Epithelial Malignancies

The signaling pathways affected by combination of proteasome and HDAC inhibitors such as the NF-κB, p53, p21^WAF1^ and JNK pathways are important for the pathogenesis of EBV-associated epithelial malignancies including NPC and gastric carcinoma. Our group reported that combination of bortezomib and SAHA could synergistically induce apoptosis of NPC cells through a ROS-dependent mechanism [61]. We also found that combination of bortezomib and SAHA could induce an enhanced acetylation of histone through a caspase-8-dependent mechanism [61]. Such caspase-8-dependent histone acetylation by combination of proteasome and HDAC inhibitors was also reported in leukemic cells [87]. However, we did not observe any up-regulation of Rb or p53 in the NPC cells in contrast to the strong up-regulation of Rb and p53 reported in other cancers upon histone hyperacetylation [89]. In addition, NF-κB inactivation was also found to be not important in the apoptosis induced by combination of bortezomib and SAHA in NPC cells [61]. The non-mitochondrial production of ROS via the nicotinamide adenine dinucleotide phosphate (NADPH) oxidase complex and endoplasmic reticulum system might be involved in the induction of apoptosis by combination of bortezomib and SAHA [90]. We further found that combination of bortezomib and several class I HDAC inhibitors (which inhibit HDAC-1, -2, -3 and -8 isoforms)), including MS-275, apicidin and romidepsin, also potently induced apoptosis of NPC cells [91]. The cell death mechanism was dependent on ROS and endoplasmic reticulum stress but independent of inhibition of HDAC6, suggesting the aggresome disruption mechanism was not involved in the cell death of EBV-positive NPC cells [91,92,93,94]. Combination of proteasome and HDAC inhibitors could also induce the up-regulation of p21^WAF1^ and down-regulation of c-myc in the EBV-positive NPC cells and other cancer cell types [79,81,82,84,95].

Combination of bortezomib and romidepsin could induce synergistic killing of gastric carcinoma cells via a summative effect of caspase-dependent apoptosis and caspase-independent autophagy [72]. Our laboratory and others had shown that the autophagic cell death induced by combination of bortezomib and romidepsin was mediated through a strong production of ROS followed by disruption of lysosomes in cancer cells [72,96,97,98,99]. Notably, combination of proteasome and HDAC inhibitors induced the generation of ROS, which was demonstrated to induce the expression of LMP1 and EBV lytic cycle reactivation, in both NPC and gastric carcinoma cells [100,101]. On the other hand, EBNA1 was also shown to induce the generation of ROS in NPC cells [102]. Thus, it would be interesting to investigate the potential interaction of between the EBV viral proteins and ROS during the killing of EBV-positive epithelial malignancies by combination of proteasome and HDAC inhibitors.

### 5.2. Combination of Proteasome and HDAC Inhibitors on EBV-Associated Lymphoid Cells

In EBV-positive lymphoid cells, EBNA-3C could downregulate the expression of tumor suppressor genes such as Bim, p53, p16^INK4A^ and p21^WAF1^ through epigenetic modification of the host cell genomes [22,45,47,49,103,104,105,106], either through interaction with C-terminal binding protein or direct recruitment of HDAC enzymes [54,107,108,109]. Because EBNA-3C could manipulate both proteasomal degradation (discussed in Section 3.2 and Section 3.3) and histone deacetylation pathways to maintain the survival of lymphoid cells, we postulated that combination of proteasome and HDAC inhibitors could act synergistically to kill the cells by counteracting EBNA-3C’s function. We showed that combination of bortezomib and SAHA induced synergistic killing of BL cells or LCLs which express EBNA-3 proteins [58]. The mechanism of killing was probably related to the up-regulation of p21^WAF1^, generation of ROS, induction of DNA damage response (DDR) and diminished G2/M arrest in the EBNA-3 expressing cells [58]. Indeed, it had been reported that induction of DDR could trigger off apoptosis in cancer cells after overriding G2/M arrest by phosphorylation of cdc25c, which could be dysregulated by EBNA-3C [60,110,111].

### 5.3. Pre-Clinical Data of Combination of Proteasome and HDAC Inhibitors on Treatment of EBV-Associated Malignancies

Bortezomib, SAHA and romidepsin are FDA-approved for the treatment of cancers [73,74,112,113]. We tested combination of bortezomib and either SAHA or romidepsin on the killing of BL, NPC and gastric carcinoma in vivo. We found that the drug combinations could synergistically suppress the growth of, BL, NPC and gastric carcinoma xenografts in nude or SCID mice [58,61,72,91]. Promisingly, bortezomib, SAHA and romidepsin could mediate strong killing on the EBV-associated malignancies at concentrations that are much lower than the clinically achievable concentrations in patients’ plasma [114,115]. The findings that the drug combinations could induce apoptosis of NPC cells in a ROS-dependent manner support the testing of potential complementary action of the drug combination to radiotherapy in the treatment of NPC patients [116]. Further testing of the in vivo anti-tumor effect of combination of proteasome and HDAC inhibitors on more EBV-associated diseases, including a subset of Wp-restricted EBV-associated BL, post-transplant lymphoproliferative disorder, diffuse large B cell lymphoma and AIDS-associated lymphoproliferative disease is warranted.

## 6. Concluding Remarks

In summary, the survival and replication of EBV in EBV-infected cancer cells require the manipulation of the host’s ubiquitin-proteasome system through the action of a myriad of viral proteins to mediate escape from immune surveillance, disruption of cell cycle regulation and suppression of apoptosis (summarized in Figure 1). Proteasome inhibitor, such as bortezomib, can counteract these viral functions and are promising agents to incorporate into novel therapeutic regimens against EBV-associated cancers. Combination of bortezomib and HDAC inhibitors represents a novel therapeutic regimen that can induce potent synergistic killing of EBV-associated lymphoid and epithelial cancers and warrants testing of its efficacy in clinical trials.

## Figures and Tables

**Table 1:** Function of Epstein-Barr virus (EBV) and Kaposi’s sarcoma-associated herpesvirus (KSHV) proteins affecting ubiquitin-proteasomal system.

Viruses	Oncogenic Proteins/Molecules Involved	Proteins Processed by UPS	Mechanisms	Cell Function Affected	References
EBV	BDLF3	MHC I & II	Postulated E3 ligase for degradation of MHC molecules is not identified yet	Immune evasion	[18]
	EBNA-1	EBNA-1	Inhibits proteasomal processing of EBNA-1 antigenic peptides	Immune evasion	[16]
		p53	Interacts with USP7, leading to ubiquitination and proteasomal degradation	Apoptosis inhibition	[33]
	EBNA-3C	pRb	Stabilizes cyclin D1/CDK6 and recruits SCF^Skp2^ E3-ubiquitin ligase to facilitate the proteasomal degradation of pRb	Cell cycle deregulation (Bypass sub-G1 arrest)	[22,23]
		p21^WAF1^	Physically interacts with Pim-1 which in turn phosphorylates p21 and enhances poly-ubiquitination of p21 for degradation	Cell cycle deregulation (Bypass G1 arrest) Apoptosis inhibition	[24]
		p27^KIP1^	Enhances the phosphorylation and proteasomal degradation of p27^KIP1^ through SCF^Skp2^ E3-ubiquitin ligase	Cell cycle deregulation (Bypass G1 & G2/M arrest)	[25]
		Bcl-6	Interacts with Bcl-6 and promotes its ubiquitination and proteasomal degradation	Cell cycle deregulation (Bypass G1 arrest) Apoptosis inhibition (release of Bcl-2)	[26]
		p53	Recruits and stabilizes MDM2 E3 ligase for proteasomal degradation of p53	Apoptosis inhibition	[34,66,67]
	LMP-1	p100	Induces proteolysis of p100 to p52 through proteasome and activates non-canonical NF-κB pathway	Apoptosis inhibition	[32]
KSHV	K/V cyclin	p27^KIP1^	Interacts with CDK6 and phosphorylates p27^KIP1^ for proteasomal degradation	Cell cycle deregulation (Bypass G1 & G2/M arrest)	[29,30]
	LANA (CR1 repeat)	N/A	Inhibits proteasomal processing of LANA antigenic peptides	Immune evasion	[17]
	RTA	IRF3 & IRF7	Promotes proteasomal degradation of IRF3 & IRF7 directly or through stabilization of RAUL	Immune evasion	[21]
	vIRF4	p53	Inhibits phosphorylation of p53 by ATM and interacts with MDM2 to facilitate proteasomal degradation of p53	Apoptosis inhibition	[35,36]

MHC, major histocompatibility complex; EBNA, EBV nuclear antigen; USP, ubiquitin-specific protease; SCF, SKP1-Cul1-F-box protein; MDM2, mouse double minute 2; LANA, latency-associated nuclear antigen; CR, central repeat; RTA, replication and transcription activator; vIRF, viral interferon regulatory factor; IRF, interferon regulatory factor; RAUL, RTA-associated ubiquitin ligase; ATM, ataxia-telangiectasia mutated.

**Table 2:** Proteasome inhibitors.

Proteasome Inhibitor	Type	Viral Protein Affected	Lytic Reactivation	Clinical Development	Structure
**Bortezomib**	Boronate	EBNA-3C (combination with SAHA or romidepsin)	EBV KSHV HSV-1	FDA-approved for MM, MCL and RRMM [39]	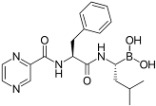
**Carfilzomib**	Epoxyketone	N.D.	EBV	FDA-approved for RRMM [117]	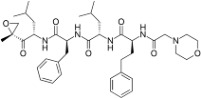
**Ixazomib**	Boronate	N.D.	N.D.	FDA-approved for RRMM Phase I clinical trials in AML, follicular lymphoma and peripheral T-cell lymphoma [118]	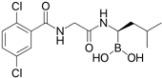
**Marizomib**	β-lactone	N.D.	N.D.	Phase I clinical trials in RRMM, solid tumors and lymphoma	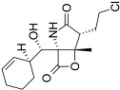
**CEP-18770**	Boronate	N.D.	N.D.	Phase I–II clinical trials in RRMM	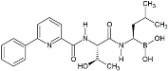
**ONX-0912**	Epoxyketone	N.D.	N.D.	Phase I clinical trials in haematological solid malignancies	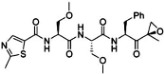

SAHA, suberoylanilide hydroxamic acid; HSV, herpes simplex virus; FDA, the Food and Drug Administration; MM, multiple myeloma; MCL, mantle cell lymphoma; RRMM, relapsed or refractory multiple myeloma; AML, acute myeloid leukemia; N.D., not determined.
